# Multiscale Coarse-Graining of the Protein Energy Landscape

**DOI:** 10.1371/journal.pcbi.1000827

**Published:** 2010-06-24

**Authors:** Ronald D. Hills, Lanyuan Lu, Gregory A. Voth

**Affiliations:** 1 Department of Chemistry, James Franck Institute and Computation Institute, University of Chicago, Chicago, Illinois, United States of America; 2 Department of Chemistry and Center for Biophysical Modeling and Simulation, University of Utah, Salt Lake City, Utah, United States of America; National Cancer Institute, United States of America and Tel Aviv University, Israel

## Abstract

A variety of coarse-grained (CG) models exists for simulation of proteins. An outstanding problem is the construction of a CG model with physically accurate conformational energetics rivaling all-atom force fields. In the present work, atomistic simulations of peptide folding and aggregation equilibria are force-matched using multiscale coarse-graining to develop and test a CG interaction potential of general utility for the simulation of proteins of arbitrary sequence. The reduced representation relies on multiple interaction sites to maintain the anisotropic packing and polarity of individual sidechains. CG energy landscapes computed from replica exchange simulations of the folding of Trpzip, Trp-cage and adenylate kinase resemble those of other reduced representations; non-native structures are observed with energies similar to those of the native state. The artifactual stabilization of misfolded states implies that non-native interactions play a deciding role in deviations from ideal funnel-like cooperative folding. The role of surface tension, backbone hydrogen bonding and the smooth pairwise CG landscape is discussed. *Ab initio* folding aside, the improved treatment of sidechain rotamers results in stability of the native state in constant temperature simulations of Trpzip, Trp-cage, and the open to closed conformational transition of adenylate kinase, illustrating the potential value of the CG force field for simulating protein complexes and transitions between well-defined structural states.

## Author Summary

Biological function originates from the dynamical motions of proteins in response to cellular stimuli. Protein dynamics arise from physical interactions that are well-predicted by detailed atomistic simulations. In order to examine large protein complexes on long timescales of biological importance, however, coarse-grained simulation approaches are needed to complement experiment. Previous coarse-grained models have proved successful for investigations involving a given protein's native structure, including protein folding and structure prediction. We construct a model capable of simulating proteins regardless of their sequence or structure. The present coarse-grained model was, however, developed rigorously from the underlying atomistic forces as opposed to knowledge-based or *ad hoc* parameterizations. Examination of the model predictions on various accessible timescales reveals successes and limitations of the model. While functionally relevant conformational transitions can be studied, the coarse-grained representation has some difficulty with the *ab initio* folding of the peptide chain into its proper structure. Our observations highlight the complex molecular nature of a protein's underlying energy landscape, offering rigorous insight into the information missing in reduced representations of the peptide chain. With these caveats in mind, the physical interaction–based, coarse-grained model will find application in simulations of a wide variety of proteins and continue to guide future coarse-graining efforts.

## Introduction

Despite continuing advances in computing power, atomistic simulation remains a considerable challenge at increasingly large time and length scales for processes of biological importance such as protein folding, conformational change and assembly. Coarse-grained (CG) approaches have therefore enjoyed popularity, in which the polypeptide can be modeled using a reduced representation of one or more, sometimes fewer, interaction sites per residue. Early CG models employed a binary code, classifying interactions between combinations of hydrophobic and polar (HP) residues [1]. HP models suffered from having a degenerate global energy minimum with as many as 10^3^ conformations. An important remaining objective therefore is the construction of a sophisticated CG potential that recapitulates the thermodynamics of the conformational landscape and identifies the native state as a stable global energy minimum consistent with energy landscape theory [2]. Such a CG potential would be of value not only to the protein folding and structure prediction communities but could prove extremely useful in general simulations of protein dynamics and conformational change.

Despite an ever-growing repertoire of independent coarse-graining approaches they still have not rivaled all-atom potentials in structure prediction [3]. Nevertheless, CG models have achieved surprising success in diverse areas of protein modeling. This success is made possible by the introduction of bias towards the native state. Elastic network models are the most restrictive example, in which backbone alpha carbons within some cutoff distance of each other in the native structure are assigned pairwise harmonic restraints. Elastic network calculations have reproduced the low frequency functional motions of a large variety of proteins [4]. Less restrictive are Gō models [5], where backbone alpha carbons in proximity in the native state are attracted via a Lennard-Jones (LJ) potential:
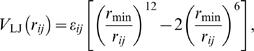
(1)in which the location and depth of the attractive minimum between particles *i* and *j* are given by *r*_min_ and *ε*, respectively. Unlike harmonic restraints, LJ interactions can spontaneously dissociate in order to visit unfolded conformations. Gō models have been successful in predicting folding mechanisms because they mimic the minimally frustrated funneled energy landscape of evolved proteins, in which non-native interactions play a minimal role [6], [7]. Lastly, general CG models have employed simple LJ interactions between all residues in the protein chain but rely on additional native dihedral or hydrogen bond restraints to counteract energetic frustration and thereby ensure structural stability during simulation of the native state [8]–[13].

Current efforts aim to improve the physical accuracy of CG nonbonded interaction schemes in order to alleviate the need for native structure restraints. Accurate CG interactions would provide for a correct description of protein dynamics and conformational change for large deviations from the native state as well as protein-protein interactions. Ongoing parameterization endeavors include various schemes. Certain empirical approaches parameterize *ad hoc* LJ functional forms using thermodynamic data such as density, surface tension, solvation energy and oil-water partition coefficients [9], [14]–[16], which have previously been used with some success in CG models of lipid bilayers [17]. Folding-inspired approaches utilize known folding behavior to tune CG parameters that will result in a properly folded protein [18]–[22]. In a somewhat similar fashion, knowledge-based methods invoke statistical potentials derived from distributions of residue-residue interactions and secondary structure in all known protein structures [3], [13], [23]–[27].

The remaining class of CG model development involves parameterization against all-atom reference simulations. Some of these approaches are based on obtaining the pairwise potentials of mean force (PMFs) between amino acid sidechains. Scheraga and colleagues have employed atomistic umbrella sampling to obtain PMFs for different packing arrangements of sidechain analog dimers [28]. Analytical approximations to the orientation-dependent, pairwise PMFs empower the UNRES model for structure prediction [29]. Another single site sidechain model for structure prediction has recently been developed by Betancourt and Omovie with pairwise PMFs obtained from atomistic simulations of all 210 amino acid pairs [30]. A model consisting of up to two interaction sites per sidechain has been developed for protein docking based on PMFs estimated from atomistic simulations of the 20 amino acid homodimers [31].

The goal of the present work is the construction of an accurate CG interaction model for the amino acids in which the packing energetics of sidechain rotamers is properly maintained using multiple interaction centers per sidechain. The CG potential is developed from forces generated from atomistic simulation, a process sometimes referred to as force matching (actually a force “renormalization”), using the multiscale coarse-graining (MS-CG) method [32]–[37]. MS-CG is a variational procedure for determining the many-body potential of mean force for the CG variables (the CG “potential”) that reproduces the equilibrium probability distribution observed in the atomistic configurational ensemble [35]. No assumptions are made about the functional forms of the pairwise interactions between CG sites [37], and multibody correlations [36] are implicitly taken into account in the resulting effective pairwise CG potential. In these respects MS-CG has a similar objective as the other multiscale methods iterative Boltzmann inversion [38] and inverse Monte Carlo [39], in which the effective pairwise potential is iteratively refined until satisfactory agreement with the atomistic radial distribution functions (RDFs) is obtained [40]. A key difference, however, is that MS-CG uses molecular scale forces as its input and not the two-body RDFs (rather, the latter is a prediction, not input, from the MS-CG model).

A challenge with multiscale methods is that the resulting CG model may be limited in applicability to the substates, or region of conformational space, sampled in the reference atomistic simulations used to construct the CG model. Previous MS-CG models have been used to accurately describe specific configurations of selected peptides [41]–[43]. In contrast, the approach used here is to apply MS-CG to a variety of peptide equilibria to obtain a general set of CG interaction potentials for the amino acids that can then be used in simulations of proteins of arbitrary sequence. The MS-CG potentials are compared and combined for atomistic simulations of the unfolded ensembles of polyalanine, polyleucine and the miniprotein Trpzip, as well as the self-association of amino acid dipeptides.

The force field is then validated by performing CG simulations of Trpzip, Trp-cage and adenylate kinase (AdK). Parallel tempering, or replica exchange molecular dynamics (REMD) [44], is used to characterize the folding energy landscapes of the three CG proteins. REMD takes advantage of simultaneous simulations at high temperature to overcome local energy barriers. Extensive sampling with CG-REMD was employed to determine the global energy minimum and illustrate potential strengths and limitations of the model. Finally, conventional constant temperature molecular dynamics (MD) is performed to demonstrate the stability of the native state and the promise of the CG force field for modeling protein dynamics.

The present work attempts to address for the first time the question of whether protein folding and dynamics can be captured with a generic reduced representation of the sidechains derived from real physical forces. In contrast to backbone centric approaches that have had some success in predicting the global minimum of helical bundles [18], [21], [22], the current sidechain centric approach appears to be more useful for simulating the native state dynamics of diverse helical and β-sheet proteins. Evaluation of our model in comparison to backbone centric models yields insight into the relative influence of the backbone and sidechains on folding and dynamics and illustrates the limitations of pairwise additive residue-level interactions in reproducing folding cooperativity.

## Methods

### CG model construction

**Figure 1:**
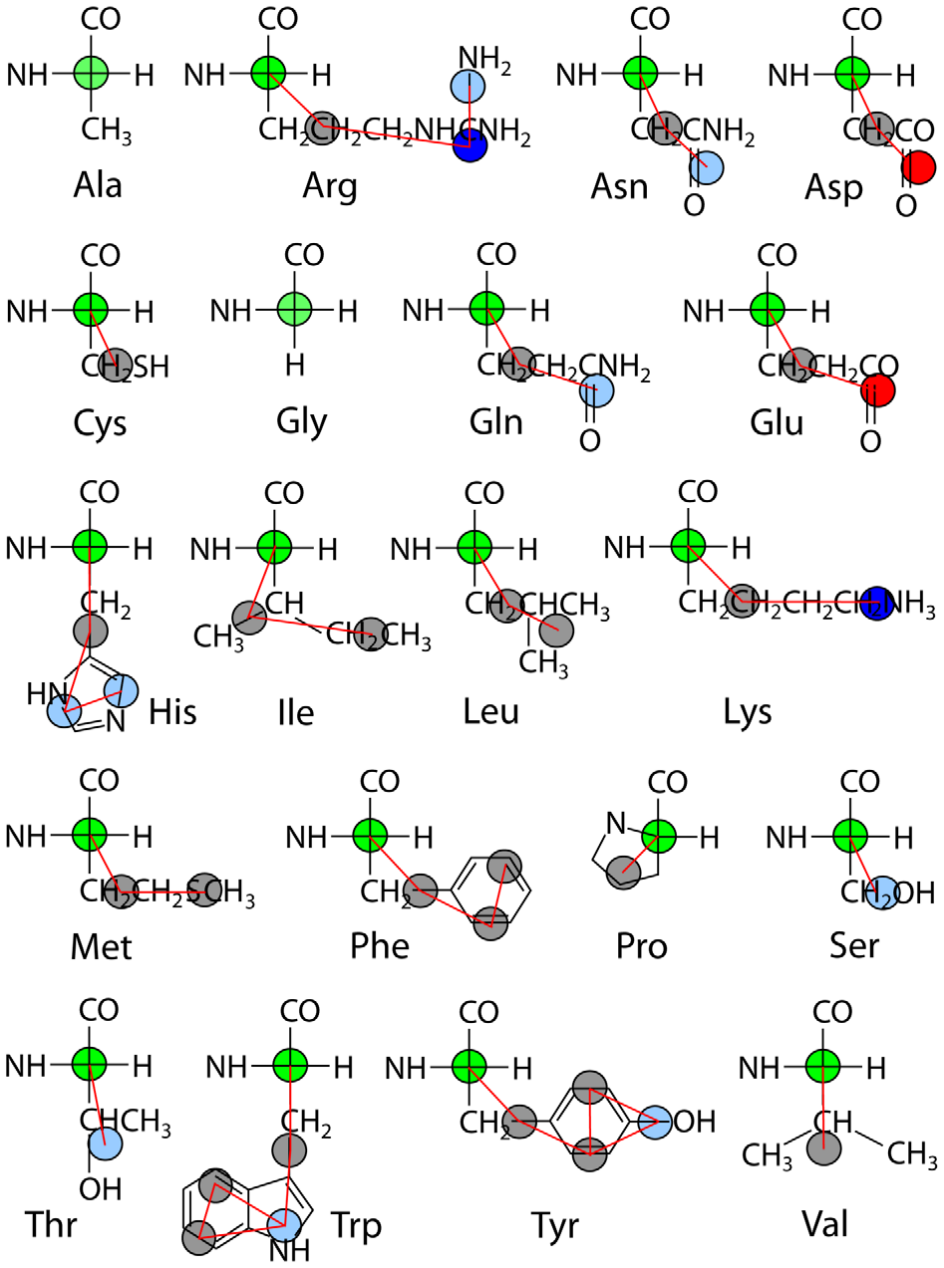
Mapping of CG site types for the amino acids. The backbone is represented by a single site at the alpha carbon (green). Sidechain sites are assigned to the mass centers of polar (light blue), apolar (gray), positive (blue) and negative (red) functional groups consisting of two or three heavy atoms. CG sites are connected by bonded interactions (red lines).

The polypeptide backbone was represented using a single CG site per residue placed at the C_α_ position in order to reasonably maintain the backbone conformational degrees of freedom [45], [46]. MS-CG was also attempted with three backbone sites per residue as in previous work on hydrogen bonding [41]–[43] but not included in the model; the resultant potentials were repulsive, likely due to the approach of averaging over all orientations present in the unfolded ensemble rather than a purely attractive native basin. MS-CG was performed by matching the instantaneous total atomistic force on the alpha carbon. As many as four CG sites were chosen for each sidechain to describe the essential orientational degrees of freedom and maintain a consistent mapping of two or three heavy atoms per site (Figure 1). MS-CG was used to determine the 15 pair potentials between combinations of five assigned CG site types (backbone, apolar, polar, positive, negative). The grouping of sidechain sites according to type was a necessary approximation in order to obtain converged pair potentials using the MS-CG algorithm. MS-CG was performed by matching the sum of the instantaneous forces on all atoms in a given sidechain site.

The solvent degrees of freedom were integrated out making this a “solvent-free” CG force field [47], [48]. No atom was involved in the definition of more than one CG site, thereby allowing for consistency in momentum space as well as configuration space between the all-atom and CG many-body PMF [35]. Absolute timescales in the CG dynamics are difficult to obtain from the model, however, due to the reduced number of degrees of freedom, lack of explicit solvent molecules and smooth energy surface.

Bonded terms were obtained directly from the atomistic distributions rather than the force matching results with MS-CG, allowing for residues to be treated uniquely regardless of site type to give a full description of steric packing. Potentials for bond lengths, bending angles and torsions in each amino acid were obtained using Boltzmann inversion:
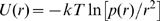
(2)

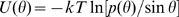
(3)

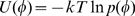
(4)where *kT* is the thermal energy and *p* is the probability distribution observed in atomistic MD; the volume normalization factors *r* ^2^ and sin *θ* were needed to properly represent all distributions. Bond and angle potentials were fit to harmonic or fourth order polynomials where appropriate; otherwise, custom bond tables were employed in GROMACS [49]. The angle from sidechain and backbone sites of residue *i* to backbone residue *i*−1 was treated separately from the angle to backbone residue *i*+1. Torsions involving sidechains were unrestricted excepting tryptophan and tyrosine rings, which were fit with improper torsions to maintain planarity. Backbone angles between three successive alpha carbons were represented using a single sequence-independent fourth order polynomial allowing rapid interconversion between α-helix and β-sheet values (see supporting Figure S1A).

Backbone torsions between four successive alpha carbons were represented using a set of sequence-dependent potentials developed from fitting the inverted distributions of known structures in the Protein Data Bank [50]. These consisted of 20^2^ fourth order cosine series, one fit for each possible permutation of the middle two residues involved in the torsion. All statistical potentials were scaled by a constant factor of 0.54, chosen to give good agreement with the polyalanine distribution from all-atom MD (Figure S2A). The inverted distribution of polyleucine in all-atom MD was well predicted by the resulting scaled statistical potential (Figure S2B). The final set of backbone torsions allowed rapid interconversion between α-helix and β-sheet, with rates generally decreasing with sidechain bulk in the order Gly∶Ala∶C_β_-branched∶Pro. Developing the bonded potentials separately from the nonbonded interactions was ultimately justified by the satisfactory agreement between bonded distributions in all-atom and CG simulations (*e.g.*, see Figures S1, S2A).

### Force matching atomistic simulations

All-atom MD was performed using the OPLS [51] protein force field with SPC solvent in the GROMACS [49] simulation package. The default parameters were employed with particle mesh Ewald for long-range electrostatics and a 1.2 nm cutoff for grid-based short-range neighbor searching. Constant NVT simulations were performed using the Nosé-Hoover thermostat with a 0.5 ps relaxation time constant. Bonds to hydrogens were constrained using LINCS and a 2 fs integration timestep was used. Each peptide system was simulated in a (4 nm)^3^ periodic box with a peptide∶water concentration of 10%. Coordinates and forces of protein atoms were recorded at intervals of 1 ps or longer for use in MS-CG force matching.

**Figure 2:**
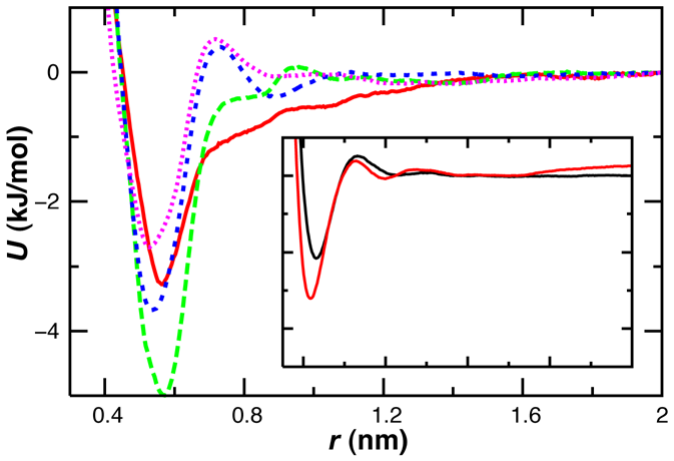
Pairwise interaction potential between nonbonded backbone alpha carbons generated from atomistic force matching. Unfolding (498 K) simulations of Trpzip (red), Leu15 (green) and Ala15 (blue) as well as 300 K MD of Ala15 (pink) yield attractive potentials that share a minimum at ∼0.6 nm. Inset (same *xy* scale): Correspondence in the interaction potentials between apolar sidechain sites for Trpzip (red) and a dipeptide solution of the 20 amino acids (black).

In order to derive a general set of CG potentials from the unfolded ensemble, peptide systems were simulated at 498 K to enhance conformational sampling. Given the modest temperature dependence of interaction potentials generated from atomistic simulation [52], [53], polyalanine is comparably shown to exhibit modest temperature sensitivity with the MS-CG scheme employed in the present work (Figure 2). Nevertheless, the higher temperature used in the simulations to define the model CG potentials will tend to “smooth out” the resulting interactions. Potentials were developed separately for different peptide systems and compared to assess the efficacy of a single potential being used independent of sequence. Ten or more independent simulations approximately 50 ns in length were performed starting from different random configurations of the following peptide systems to yield a composite simulation length of at least 0.5 µs for each system (Table 1). Five Ala15 peptides were simulated in a box of water molecules at 300 K as well as 498 K. Three unfolded Leu15 peptides were simulated in one water box. Three molecules of the miniprotein Trpzip2 [54] were simulated in another water box. Lastly, 25 dipeptides were randomly placed in a single water box, one for each amino acid with the exception of two for alanine, glycine and lysine and three for aspartate. To ensure ample exchange between sidechain association partners and convergence in the developed MS-CG potentials, 200 independent simulations of the dipeptide solution were performed for a composite length of 9 µs. A few remaining bonded terms were obtained from distributions in all-atom unfolding simulations of two Trp-cage5b [55] proteins in water and five AACHMFVAA peptides in water, although neither system was force-matched. A single natively structured Trpzip molecule was also simulated in water at 300 K for comparison of structural fluctuations between all-atom MD and CG-MD with the final model. Excepting the dipeptides, each terminus was uncapped and charged. Unfolded starting configurations were generated by randomly orienting the peptides in Cartesian space and then equilibrating for 2 ns at 700 K; the random number generator was employed with varying seeds.

**Table 1:** Peptide systems employed in all-atom reference simulations.

Sequence	*T* (K)	Peptides^a^	Total µs
Ala15	300^b^, 498^b^	5, 5	1.7, 0.6
Leu15	498^b^	3	0.5
Trpzip	300, 498^b^	1, 3	1.2, 0.6
Trp-cage	498	2	0.5
dipeptide solution	498^b^	25	9.2
AACHMFVAA	498	5	0.6

a Number of peptides placed in the (4 nm)^3^ water box.

b System was used for force matching nonbonded interactions. Bonded interactions were obtained from all systems via distribution fitting.

The force matching of the atomistic reference simulations in the MS-CG method was performed using the program MSCGFM [56], a fast and flexible implementation of MS-CG. A linear spline basis set was employed for least squares optimization of nonbonded interaction pairs separated by less than 2 nm and more than three bonds. The computation was made feasible by employing the block-averaging procedure of combining separate solutions for disjoint sets of configurations (blocks) [37]. Blocks consisted of 2,000 or more frames for each peptide system depending on memory requirements. The resulting pairwise force curves were integrated and smoothed using a cubic B-spline to obtain tabulated potentials for input in GROMACS. Repulsive positive-positive and negative-negative interactions were switched linearly to zero over the range 1 nm to 1.2 nm, resulting in ∼1 kJ/mol error over the switching region. Convergence of the nonbonded interactions was checked by repeating the MS-CG calculation with half the configurations and ensuring the force curves were similar excepting high frequency noise.

### CG molecular dynamics

CG simulations were performed in GROMACS 4 [49] using Langevin dynamics with a 2 ps inverse friction constant to maintain thermal equilibrium and a 2 fs integration timestep. Tabulated nonbonded interactions were updated every step and calculated between all CG sites separated by at least three bonds using a 1.2 nm distance cutoff. A single set of 15 site-site CG potentials, chosen as most representative across peptide systems, was employed for all CG proteins.

CG-MD of polyalanine was performed on five copies of Ala15 placed in a 40 Å periodic box for 50 ns starting from a random configuration obtained from the endpoint of a 58 ns atomistic MD run at 498 K described above. Polyalanine aggregation was monitored by computing the pairwise RDF of inter- and intramolecular alpha carbons separated by at least three bonds. Native state CG-MD simulations of Trpzip, Trp-cage and the open and closed forms of AdK were begun from NMR structures 1LE1.pdb [54] and 1L2Y.pdb [55] and crystal structures 4AKE.pdb and 1AKE.pdb, respectively, and performed for 200 ns at 0.6 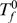, where 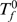 is the reference temperature for the CG model. The reference temperature was defined as the folding transition, or melting, temperature observed in CG-REMD folding simulations of Trpzip.

CG-REMD folding simulations were performed with exponentially spaced temperature replicas spanning 100–700 K. The number of replicas was chosen to maintain an exchange frequency between 20% and 40% throughout the simulation and was first estimated using *P*_des_ [57]. CG-REMD of Trpzip and Trp-cage required 16 replicas while AdK required 48 replicas. Conformational exchanges between temperature windows were attempted and snapshots recorded for Trpzip/Trp-cage every 200 ps and for AdK every 20 ps. For Trpzip and Trp-cage two independent simulations of 3 µs or longer were performed starting from an extended structure to bring the total simulation length to 6 µs per replica. To check convergence 3 µs was then performed starting from the native state. Trp-cage simulations converged to a common structure. Two additional simulations were performed with it as the starting point to verify the global minimum. For the more complex folding landscape of AdK, four independent simulations were performed for 80 ns, each starting from extended, closed, open or a 50/50 mixture of open and extended states. Extended starting structures were generated from equilibration in CG-MD at 700 K for at least 10 ns.

Folding landscapes were characterized by computing the root mean square deviation (RMSD) from the native structure of replica conformations corresponding to 0.6 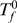. The RMSD from the CG representation of the native state was computed for a subset of backbone or sidechain sites after superimposing the backbone alpha carbons of the region of interest. CG-REMD was also performed with position restrained backbone alpha carbons starting from the native structure to determine the distribution of sidechain rotamers (3 µs for Trpzip/Trp-cage and 80 ns for open and closed AdK). The mean and standard deviation of the RMSD of sidechain sites from the native structure were computed over the fixed backbone CG-REMD simulations as well as the unrestrained CG-MD, both at 0.6 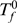. CG conformations were visualized using VMD [58].

## Results

### Transferable CG interactions

The most significant approximations used in construction of the present CG model are threefold. Atomistic reference simulations were performed at elevated temperature to denature the protein ensemble and allow for rapid interchange between association pairs in aggregated peptides. Secondly, site-site interaction potentials were obtained from different peptide systems in order to obtain a single force field applicable across protein sequences. Lastly, the through-space interactions constitute an average over the chemical diversity of the amino acids grouped into five CG site types. These assumptions enabled the construction of a versatile model for simulating proteins of arbitrary sequence and conformation.

Overall, a reasonable correspondence was observed between the CG potentials developed separately from different peptide systems for a given interaction. Figure 2 shows the similarity in C_α_-C_α_ and apolar-apolar interactions across peptide sequences. For each of the 15 nonbonded interaction pairs in the model the MS-CG potential was employed that was most representative across peptide systems. The one exception is the case of positive-negative salt bridges. Force matching of Trpzip and the dipeptide solution yielded potential minima of −10 kJ/mol and −37 kJ/mol, respectively, likely due to the influence of the hydrophobic environment; the former was adopted in the CG model to avoid large forces. Figure 2 also illustrates that the temperature dependence of the developed CG potential for polyalanine is comparable in magnitude to the variation between different sequences. The modest temperature dependence of CG potentials obtained from atomistic data has been noted previously [52], [53]. The final set of CG potentials employed (Figure S3) have profiles similar to atomistic PMFs obtained at room temperature [28]. When arranged according to strength and location the attractive minima follow an expected relationship to polarity (Table 2).

**Table 2:** Location and depth of attractive minima in nonbonded interactions between CG site types.

Interaction Pair	*r*_min_ (Å)	*ε* (kJ/mol)^a^
positive-negative	3.2	9.6
C_α_-C_α_	5.4	3.6
apolar-apolar	4.4	3.2
C_α_-positive	4.8	2.4
C_α_-polar	4.4	1.9
polar-polar	4.2	1.7
apolar-polar	4.4	1.6
C_α_-apolar	4.8	1.4
polar-positive	4.0	1.1
C_α_-negative	4.6	0.1^b^
polar-negative	5.1	0.1^b^
apolar-negative	5.8	0.1^b^
apolar-positive	6.5	0.1^b^
positive-positive	5.5	0.1^b^
negative-negative	5.5	0.1^b^

a Each tabulated potential had a softer core repulsion [16] than the LJ function with the same minimum.

b Potential was predominantly repulsive. See Figure S1.

### Role of CG temperature

**Figure 3:**
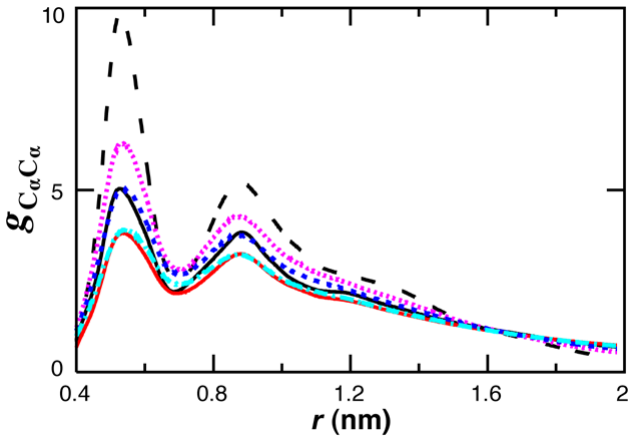
Temperature correspondence in CG-MD simulations of polyalanine aggregation. The RDF between nonbonded alpha carbons is shown for CG simulations at 300 K (black dash), 498 K (pink), 600 K (blue) and 700 K (cyan) and atomistic simulations at 300 K (solid black) and 498 K (red).

As is well appreciated from atomistic MD studies with continuum solvent, surface tension can be an elusive property in implicit solvent models and is often approximated as a simple function of the solvent accessible surface area [59]. Typically, CG potentials for protein folding are scaled by a constant factor so that *T*_f_ matches experiment [50]. Simulations with the present CG model exhibited a higher effective surface tension than atomistic simulations at the same temperature, as evidenced by a greater tendency to aggregate. As inferred from CG-MD of polyalanine aggregation, increasing the temperature can reproduce the pairwise RDF of nonbonded alpha carbons from atomistic MD (Figure 3). In the statistical mechanical framework of MS-CG temperature corrections should not be needed [35], and their use may reflect inadequacies in the pairwise CG potential or basis set employed in the optimization [36], [60]. Excessive peptide aggregation with pairwise CG PMFs has been reduced elsewhere by the inclusion of explicit waters [61], [62]. The high sampling temperature, peptide concentration and sequences of the atomistic reference simulations are also sources of error in the developed CG potential.

**Figure 4:**
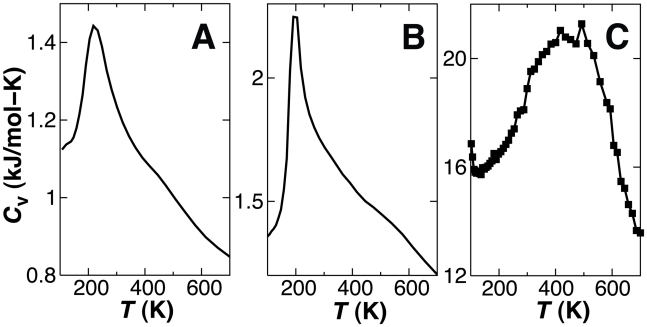
Heat capacity as a function of temperature for CG-REMD folding simulations of Trpzip (A), Trp-cage (B) and AdK (C). The reference temperature for the CG model is taken to be the folding transition temperature observed for Trpzip (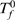), defined by the maximum in *C*_v_(*T*). Line and data point thickness denote error.

To determine the appropriate temperature range for protein simulations using the CG model the temperature dependence of the heat capacity was computed for Trpzip, Trp-cage and AdK from CG-REMD folding simulations (Figure 4). Unfolding transitions occurred at temperatures as low as 200 K. CG simulations of the native state were therefore performed at 0.6 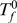, where 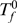 is the folding transition temperature defined by the maximum in the heat capacity observed in Trpzip simulations. CG Trpzip/Trp-cage exhibited transition temperatures of 218 K/198 K, equal in ratio to their experimental melting temperatures 345 K/315 K [54], [55]. The designed miniprotein Trpzip has an experimental melting temperature typical of natural proteins and was therefore used to define the reference temperature of the CG model. Since the CG potential is less “rough” than the actual atomistic potential, it is perhaps not surprising that a lower temperature is required for the CG protein simulations in order to effectively compensate for this feature of the model. Nevertheless, this aspect of the modeling is not completely satisfactory and will therefore be a focus of future improvements in the methodology and CG model.

### CG-REMD folding landscapes

Parallel tempering was used to characterize the CG energy landscape. Performing REMD over a wide temperature range (100–700 K) starting independently from unfolded as well as native states enabled near-canonical sampling of low energy conformations, some of which were non-native as judged from structural RMSD. Ensemble simulations were used to evaluate the accuracy of the force field in identifying the native structure as the global energy minimum.

**Figure 5:**
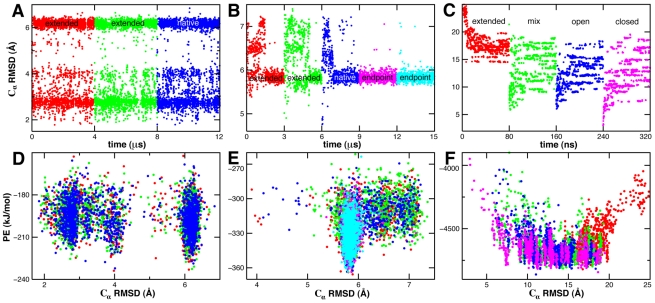
CG-REMD folding landscapes collected at 0.6 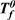. The C_α_ RMSD from the native state is shown versus composite simulation time (A–C) or potential energy (D–F) for independent simulations (different colors) of Trpzip (A,D), Trp-cage (B,E) and AdK (C,F). Simulations were started from extended and native structures as well as their common final configuration in the case of Trp-cage. AdK simulations were started from extended, closed, open and a mixture of open and extended states; RMSD is computed from the closed crystal structure. Large structural changes between snapshots, such as folded to misfolded transitions, correspond to conformational exchanges between replicas of similar energy.

The sampling convergence of CG-REMD folding simulations can be seen in Figure 5. Trpzip simulations starting from the unfolded state (7.1 Å C_α_ RMSD from native) rapidly convert to and exchange between three stable native-like conformations (Figures 5A,D). The conformation with 2.5 Å C_α_ RMSD consists of a proper β-hairpin backbone, though the Tryptophan zipper occurs on the wrong side of the β-sheet (Figure S4A). The 4 Å and 6 Å C_α_ RMSD conformations contain the Trp zipper on the correct side of the β-hairpin but allowed for distortions in the backbone of varying degrees (Figure S4A). Trp-cage simulations starting from the unfolded state (6.4 Å C_α_ RMSD from native) converge to a stable global minimum with 5.8 Å C_α_ RMSD (Figures 5B,E). The global minimum resembles the native helix-coil motif, albeit with a distorted helix (Figure S4B).

In contrast to 12-residue Trpzip and 20-residue Trp-cage, the CG energy landscape of 214-residue AdK is indicative of a frustrated random heteropolymer. Conformations with C_α_ RMSDs spanning the range 7–19 Å were visited with equal frequency once simulations starting from different initial structures converged (Figures 5C,F). The non-two-state nature of AdK's glassy folding transition is underscored by the lack of a sharp melting transition in the heat capacity curve (Figure 4). Such deviations from the funneled landscape attributed to evolved proteins [2] emphasize the role of non-native interactions (contacts not present in the native state), whose repulsive nature must be underestimated in the coarse-grained representation. The high degeneracy of AdK's global energy minimum compared to Trpzip and Trp-cage is likely due to its large domain size and vast number of possible backbone conformations.

### Native state is stable in CG-MD

**Figure 6:**
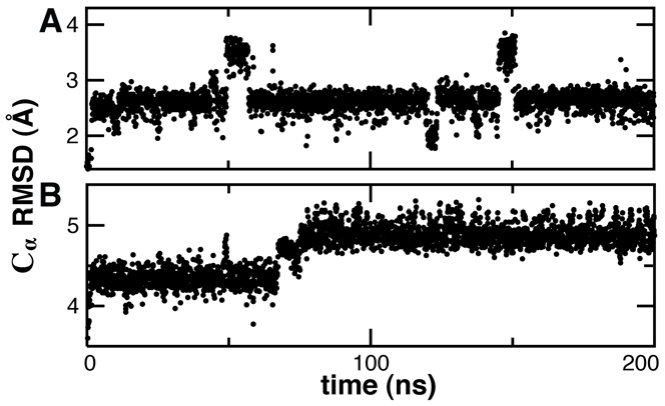
Native state CG-MD at 0.6 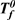. The instantaneous C_α_ RMSD from the native state is shown for simulations starting from the native structure for Trpzip (A) and Trp-cage (B). By comparison, all-atom MD of Trpzip at 300 K yielded a mean C_α_ RMSD of 0.8±0.2 Å.

**Figure 7:**
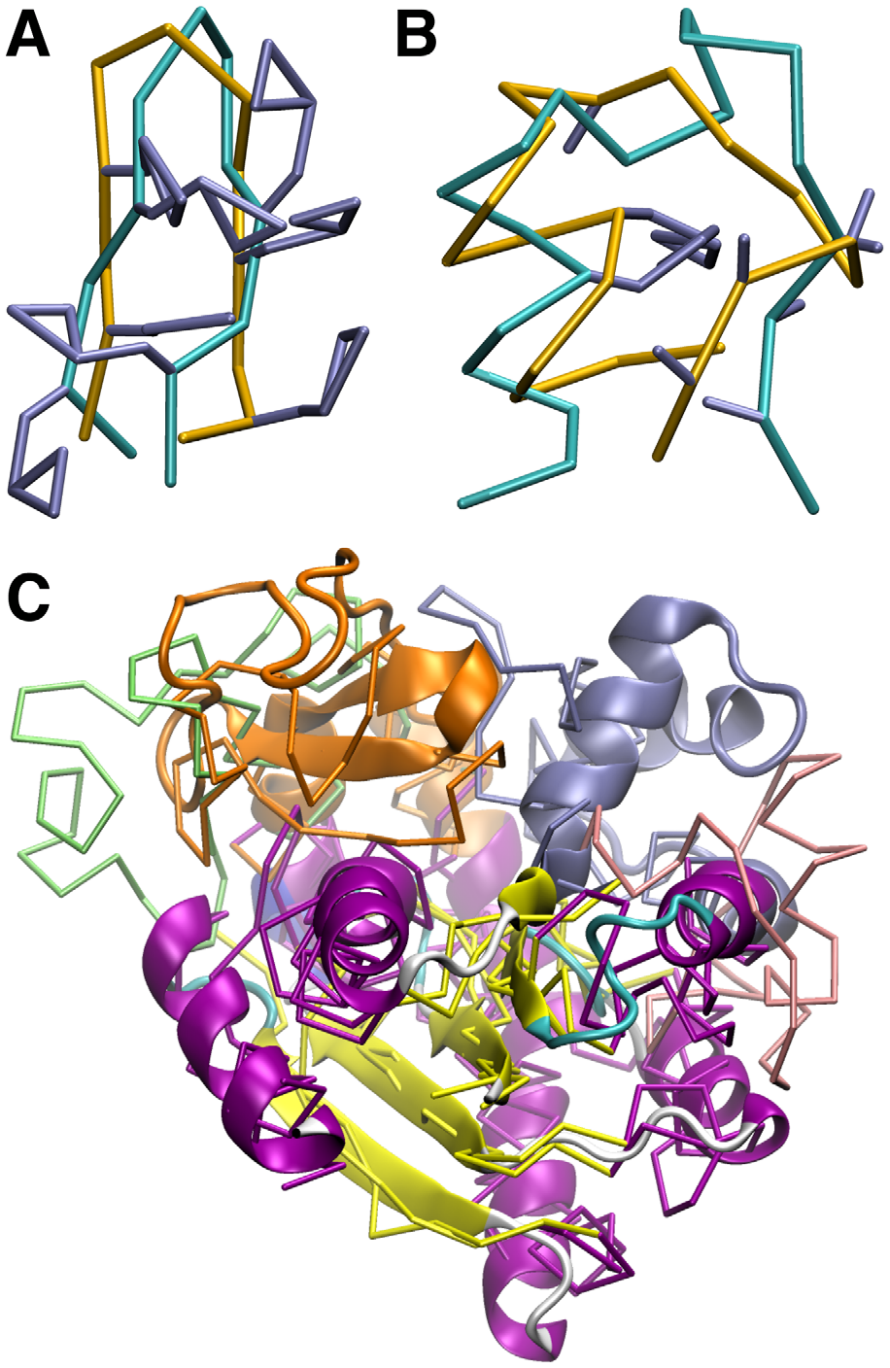
Backbone C_α_-trace structures at the end of native state CG-MD at 0.6 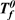 for Trpzip (A), Trp-cage (B) and AdK (C). Starting native structures are shown in cyan for Trpzip/Trp-cage and as a ribbon diagram for closed AdK. Tryptophan and proline sidechains are shown in blue (A,B). The LID/NMP domains of the AdK endpoint conformations are colored green/pink for simulations starting from open AdK and orange/blue for simulations starting from closed AdK.

Successes and failures in *ab initio* folding notwithstanding, the goal of the present work was the construction of a CG force field for modeling proteins in known structural states. Conventional constant temperature simulations were therefore performed at 0.6 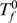 to assess the stability of the native state under CG-MD. Trpzip and Trp-cage exhibited structural stability with final configurations of 2.6 Å and 4.7 Å C_α_ RMSD, respectively, from the starting native structure after 200 ns of CG-MD (Figure 6). A slight bimodal distribution was observed in RMSD, but this was mainly due to fraying in the residues at the N- and C-termini (Figures 7A,B).

### Conformational transition in AdK

Atomistic simulation was recently used in conjunction with umbrella sampling to characterize the oft-studied open to closed conformational transition of AdK [63], in which the LID and NMP domains undergo a 14 Å relative hinge bending motion about the CORE domain. The study suggested in the absence of ligand AdK fluctuates about the open crystal structure, occasionally visiting conformations near the closed crystal structure. Binding of an adenosine polyphosphate substrate analog to the arginine-lined active site was observed to dramatically stabilize the closed conformation. To examine the suitability of the current CG force field for studying conformational transitions, CG-MD was performed for 200 ns at 0.6 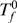 starting from both the open and closed AdK structures.

**Figure 8:**
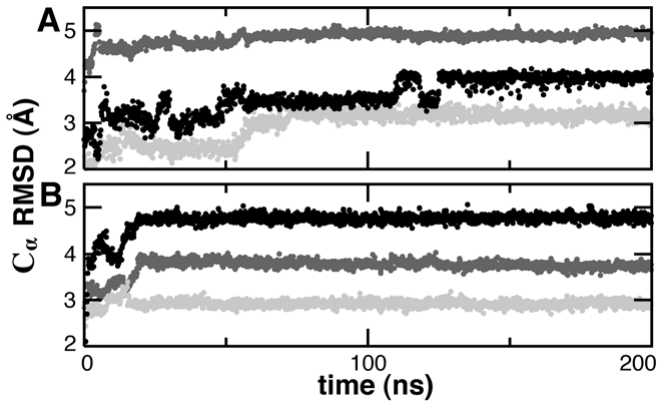
Native state CG-MD at 0.6 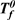 starting from the open (A) and closed (B) forms of AdK. The instantaneous C_α_ RMSD from the starting structure is shown for the CORE (dark gray), LID (light gray) and NMP (black) domains.

**Figure 9:**
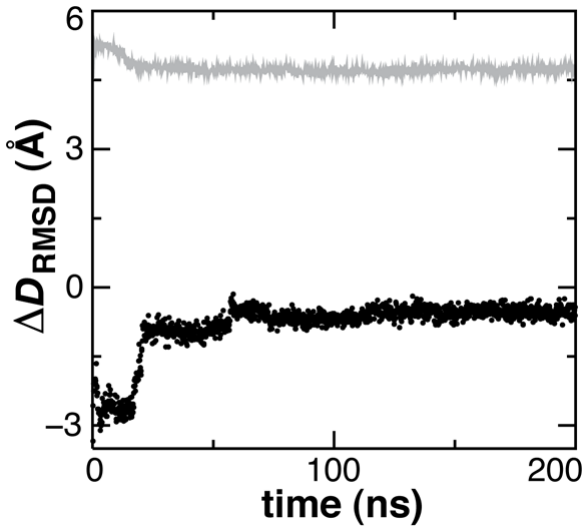
Conformational transition in AdK expressed as the difference in RMSD from the open and closed states. Simulations starting from the open structure (black circles), Δ*D*_RMSD_ = −7 Å, are seen to partly converge toward simulations starting from the closed structure (gray trace), Δ*D*_RMSD_ = +7 Å.

The CORE, LID and NMP domains are stable in CG-MD of both the open and closed states (Figure 8), each of which individually has a structural RMSD between the open and closed crystal structures of less than 2 Å when domains are superimposed. The open to closed conformational transition was monitored in AdK simulations using the reaction coordinate Δ*D*_RMSD_ [63], defined as the RMSD of backbone and sidechain sites from the open state minus their RMSD from the closed state. The simulation of the closed conformer undergoes limited structural rearrangement (4.6 Å final C_α_ RMSD to starting structure) compared to the simulation starting from the open conformer (8.0 Å final C_α_ RMSD to starting structure). Indeed, values of the reaction coordinate approach positive Δ*D*_RMSD_ (become more closed-like) in simulations of the open state (Figure 9).

The dynamics of AdK in CG-MD can be understood in terms of surface tension. Just as polyalanine exhibited an exaggerated surface tension for a given temperature, Trpzip, Trp-cage and AdK are more compact than the native structure under low temperature folding conditions in CG simulations (Table 3). Even under unfolding conditions, the peptide chains are disordered in compacted globules up to temperatures exceeding 500 K (Table 3), as can also be seen by the long tails in the heat capacity curves up to 700 K (Figure 4). Under CG-MD, the open state of AdK rapidly adopts a more compact and stable conformation that is structurally similar to the closed crystal structure, though the LID and NMP domains are not in contact. The simulation starting from the closed state also adopts a compacted structure in which the LID and NMP domains are in closer contact (Figure 7C).

**Table 3:** Distributions of radius of gyration in folding simulations.

Protein	*T* (K)	<*R*_g_/ 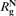 >^a^	σ	Resolution^b^
Trpzip	148	0.86	0.02	CG
Trp-cage	148	0.86	0.01	CG
AdK^closed^	139	0.91	0.01	CG
*Trpzip*	*300*	*0.97*	*0.02*	*AA*
Trpzip	366	0.98	0.05	CG
Trp-cage	366	0.98	0.04	CG
AdK^closed^	361	0.97	0.03	CG
*Trpzip*	*498*	*1.16*	*0.17*	*AA*
*Trp-cage*	*498*	*1.28*	*0.20*	*AA*
Trpzip	540	1.10	0.13	CG
Trp-cage	540	1.12	0.14	CG
AdK^closed^	546	1.77	0.33	CG

a Radius of gyration is shown relative to that of the native structure (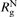).

b CG-REMD simulations are shown compared to *all-atom (AA)* reference simulations at selected temperatures.

Besides surface tension, other possible explanations exist for the incorrect relative arrangement of the LID and NMP domains during CG simulations. The negatively charged substrate needed to counteract repulsion in the arginine-lined binding pocket is absent from the simulations. Experimentally, the LID and NMP domains exhibit reduced thermodynamic stability compared to the CORE domain [64]. The open to closed conformational transition requires many subtle backbone rearrangements in the hinge regions connecting the three domains [64]–[66]. Lastly, an alternative explanation is that the reduced bulk of the low resolution interaction sites in the CG model fails to fully account for the effect of the underlying atomistic steric clashes. Structural compaction in peptide coarse-graining has been reported previously [42]. The reduced temperature used in the simulations could also be a contributor to structural compaction.

In the case of large conformational transitions in which the rearrangement can be viewed as a local refolding event [64], [66], the CG force field could potentially benefit from the addition of a loose elastic network to maintain the backbone topology analogous to previous work [10], [67]. Backbone restraints could also be used in order to predict sidechain configurations for low resolution experimental structures in which only the backbone C_α_ positions are known. Indeed, parallel tempering of Trpzip, Trp-cage and AdK with fixed native backbone topology yielded improved distributions of native sidechain configurations (Table 4, Figure S5). The fact that sidechain packing is reproduced to within 3 Å RMSD suggests that the CG description constitutes a reasonable representation of sidechain sterics and polarity, although higher resolution models [15], [68] are expected to improve accuracy.

**Table 4:** Distributions of sidechain RMSD in native state simulations at 0.6 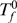.

Domain	Fixed backbone<RMSD^SC^>	σ_RMSD_	Unrestrained<RMSD^SC^>	σ_RMSD_
Trpzip^b^	3.5	0.3	6.6	0.5
Trp-cage	3.6	0.2	6.8	0.8
CORE^open^	3.0	0.2	6.0	0.3
LID^open^	2.9	0.2	5.0	0.4
NMP^open^	2.9	0.2	5.2	0.4
CORE^closed^	2.9	0.1	5.0	0.2
LID^closed^	3.3	0.2	4.4	0.1
NMP^closed^	2.9	0.2	6.6	0.3

a The RMSD (Å) of sidechain (SC) sites from the native structure was monitored in native state simulations with fixed backbone (CG-REMD) or no restraints (CG-MD).

b By comparison, unrestrained all-atom MD of Trpzip at 300 K yielded a mean sidechain RMSD of 2.5±0.5 Å.

## Discussion

A CG force field for the amino acids was developed based on microsecond all-atom simulations of peptide folding and association. Previously, the accuracy of CG functions has been assessed based on their ability to identify the native state as lower in potential energy than decoy structures [14], [25], [27], [30]. The present CG model was evaluated based on analysis of folding energy landscapes generated from REMD simulations. Non-native structures were observed with energies similar to that of the native state, which is in accord with replica exchange investigations of other CG representations for folding [18] and structure prediction [69]. Deviations from the funneled landscape indicate that the smooth landscape of CG interactions may fail to capture the effective repulsion between non-native contacts in the rugged atomistic landscape. The current sidechain centric model emphasizes sequence at the expense of detailed backbone hydrogen bonding, both of which in conjunction have been shown to determine the tertiary structure of proteins [70]. At the other end of the spectrum, backbone centric models contain three or more backbone interaction sites per residue to incorporate geometric hydrogen bond constraints at the expense of sidechain rotamers, which are represented by a singe site at the beta carbon position [18], [21], [22]. Backbone centric models have successfully predicted the structure of certain α-helix bundles excepting topological degeneracy. In contrast, the current sidechain centric approach consisting of a single site per backbone and multiple sites per sidechain was demonstrated to be more useful in simulating the dynamics of diverse helical and β-sheet proteins.

The limited success of pairwise alpha carbon interactions in folding prediction can be attributed to the fact that pairwise additive interactions at the residue level are not adequate to describe the highly cooperative process of protein folding [71]. C_α_ Gō models, for instance, require the introduction of either a desolvation barrier [72] or native dihedral backbone angular restraints to ensure a cooperative folding transition [7]. Desolvation of neighboring water molecules can be considered a multibody effect, as can the angular dependence of backbone hydrogen bonding. That the pairwise potentials developed and tested in the present work lack an appreciable desolvation barrier (Figures 2, S3) offers an additional explanation for their limited success in folding prediction.

Misfolded structures have also been observed with high probability in atomistic folding studies employing implicit solvent models [59], [73]–[75], suggesting surface tension and solvation effects are critical in reproducing the energy landscape of proteins. With current computational resources, the ability of modern all-atom force fields to capture the energy landscape can now be assessed in explicit solvent simulations using replica exchange methods [76], [77]. Obtaining the delicate balance between α-helix and β-sheet energetics is challenging, but ongoing all-atom efforts are showing promise [78], [79].

Whether a single CG force field is capable of reproducing the full thermodynamic landscape of structurally diverse proteins remains a difficult question. A variety of useful CG models do exist for studying protein folding mechanisms [7] and structure prediction [3]. The present work describes a general CG force field derived from molecular-scale interactions that is capable of stable native state simulations without the need for additional structural restraints, an improvement over existing CG models. Improved structural stability can be attributed to the explicit treatment of sidechain rotamers, their steric packing and energetics, resulting in the native state being a local energy minimum. Future refinements of the model to better describe backbone hydrogen bonding are expected to improve its performance. However, the current force field may also prove useful in the modeling of protein complexes and their transitions.

## Supporting Information

Figure S1
**Backbone angle distributions.** (A) The inverted probability distributions are shown for all individual angles between three successive alpha carbons in all-atom MD of all proteins studied: Ala15, Leu15, Trpzip and Trp-cage. (B) The inverted distribution is shown for each backbone angle in Trpzip under CG-REMD at 0.6 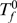. Angles corresponding to α-helix (red) or β-sheet (black) structures could be weakly stabilized with equal probability. A single fourth order polynomial potential (green) was employed for all backbone angles in the CG model independent of sequence.(0.32 MB TIF)

Figure S2 **Backbone dihedral distributions.** (A) The inverted probability distribution is shown for the pseudodihedral angle between four successive alpha carbons in all-atom MD of Ala15 at 300 K (black) and 498 K (green). Sequence dependent statistical potentials derived from the PDB were scaled by a constant factor so that good agreement is obtained for polyalanine (pink). The result from CG-MD of Ala15 is shown in blue. (B) The inverted backbone pseudodihedral distribution of polyleucine in all-atom MD (black) is well predicted by the scaled statistical potential (pink).
(0.29 MB TIF)

Figure S3 **Tabulated nonbonded interaction potentials employed between CG site types.** (A) Five potentials are shown for alpha carbons paired with the five site types: alpha carbon (green), apolar (black), polar (cyan), positive (blue) and negative (red). (B) Similarly, potentials are shown for apolar sites paired with four site types: apolar (black), polar (cyan), positive (blue) and negative (red). (C) Potentials are shown for polar sites paired with site types: polar (cyan), positive (blue) and negative (red). (D) Lastly, potentials are shown for three site pairings: positive-positive (blue), positive-negative (red) and negative-negative (pink). All 15 potentials have softer core repulsions than an LJ potential. Model parameters are available upon request.
(0.48 MB TIF)

Figure S4
**Energy minima in CG-REMD folding simulations.** Representative snapshots are shown corresponding to the three energy minima of Trpzip (A) and the global minimum of Trp-cage (B), along with their C_α_ RMSD from the native structure. Backbone C_α_-traces are shown in yellow along with Trp, Pro and other sidechains in blue. The Trp-cage native structure is shown in cyan (backbone). Snapshots were obtained from CG-REMD folding simulations collected at 0.6 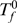 (see Figure 5).(1.99 MB TIF)

Figure S5
**Sidechain prediction with CG-REMD.** The final configuration is shown after 80 ns of CG-REMD at 0.6 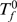 with the backbone (yellow) fixed in open AdK. Sidechain sites (blue) exhibited final RMSDs of 2.9 Å, 3.1 Å and 2.9 Å from their starting native positions (cyan) for the LID (A), CORE (B) and NMP (C) domains, respectively.(2.35 MB TIF)
