# Supplementary figures and images for: Multiscale Coarse-Graining of the Protein Energy Landscape
Source: PLoS Comput Biol. 2010 Jun 24;6(6):e1000827. doi: 10.1371/journal.pcbi.1000827 (PMC2891700; doi:10.1371/journal.pcbi.1000827)

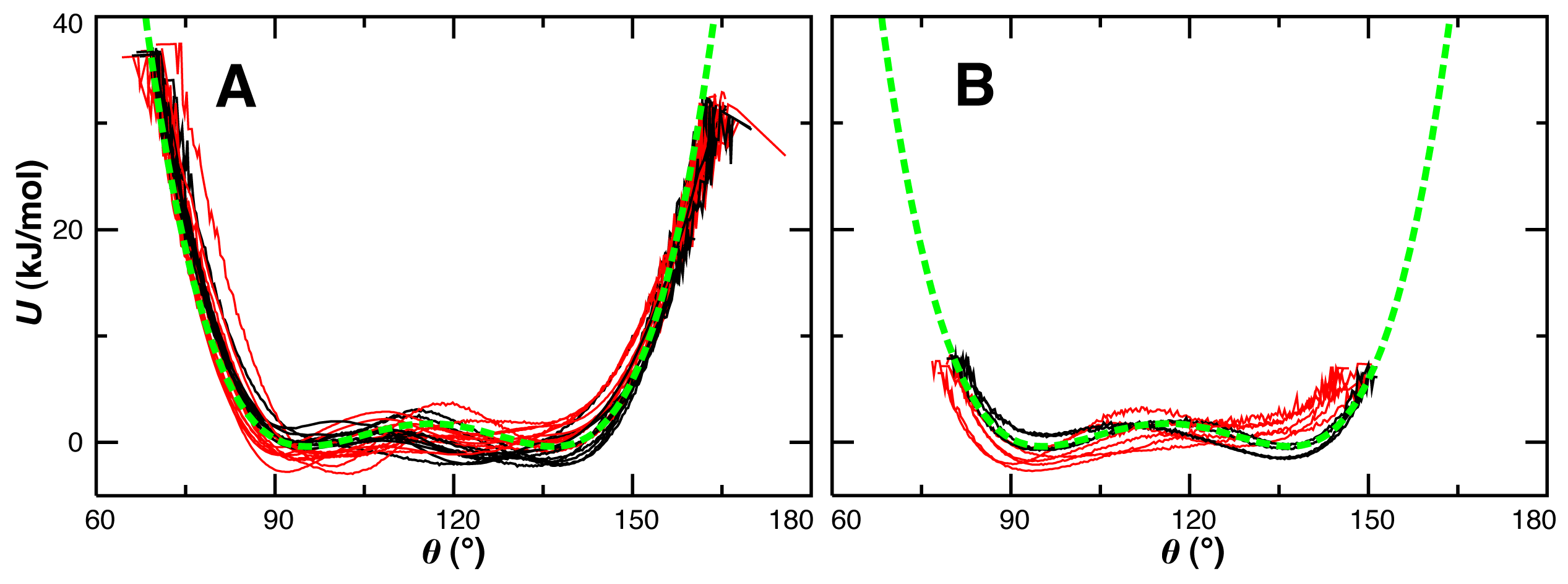

Supplement: Figure S1 — Backbone angle distributions. (A) The inverted probability distributions are shown for all individual angles between three successive alpha carbons in all-atom MD of all proteins studied: Ala15, Leu15, Trpzip and Trp-cage. (B) The inverted distribution is shown for each backbone angle in Trpzip under CG-REMD at 0.6 . Angles corresponding to α-helix (red) or β-sheet (black) structures could be weakly stabilized with equal probability. A single fourth order polynomial potential (green) was employed for all backbone angles in the CG model independent of sequence. (0.32 MB TIF) [file pcbi.1000827.s001.tif]

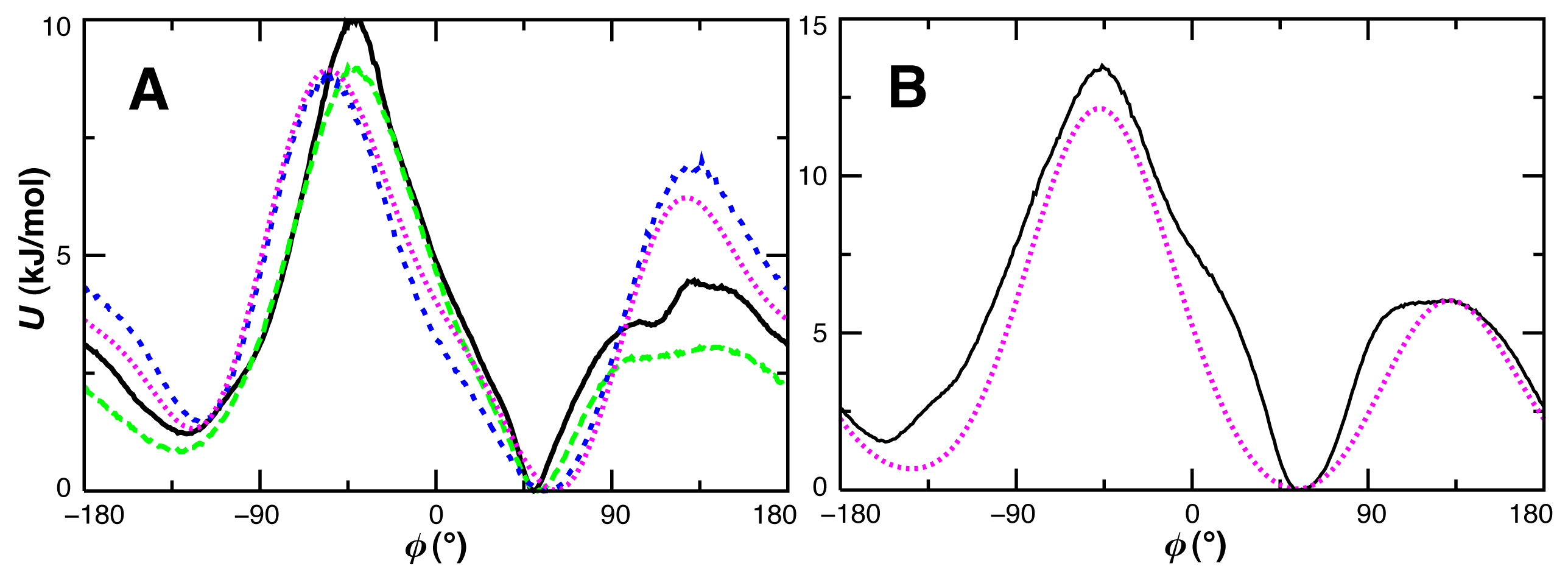

Supplement: Figure S2 — Backbone dihedral distributions. (A) The inverted probability distribution is shown for the pseudodihedral angle between four successive alpha carbons in all-atom MD of Ala15 at 300 K (black) and 498 K (green). Sequence dependent statistical potentials derived from the PDB were scaled by a constant factor so that good agreement is obtained for polyalanine (pink). The result from CG-MD of Ala15 is shown in blue. (B) The inverted backbone pseudodihedral distribution of polyleucine in all-atom MD (black) is well predicted by the scaled statistical potential (pink). (0.29 MB TIF) [file pcbi.1000827.s002.tif]

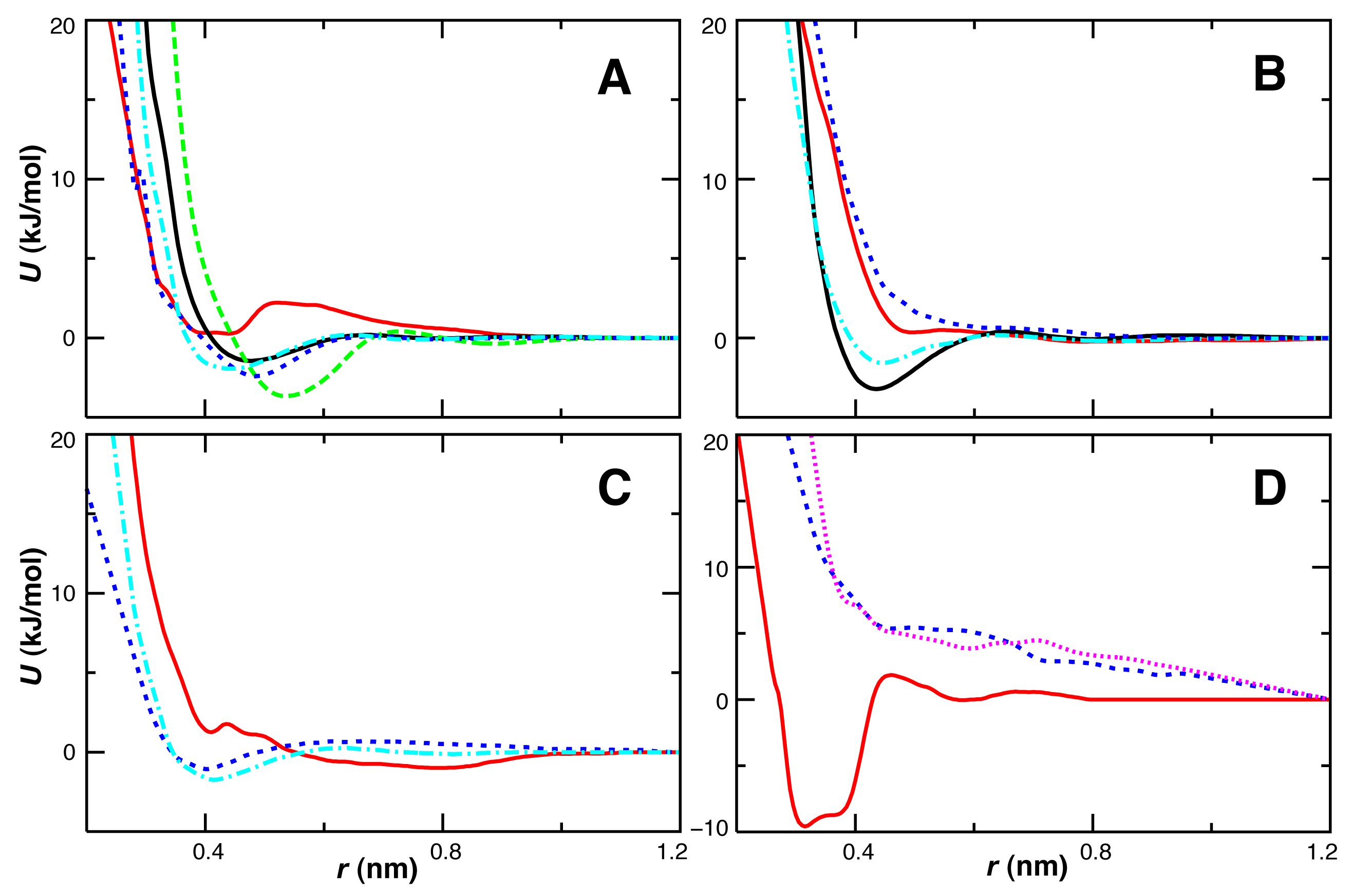

Supplement: Figure S3 — Tabulated nonbonded interaction potentials employed between CG site types. (A) Five potentials are shown for alpha carbons paired with the five site types: alpha carbon (green), apolar (black), polar (cyan), positive (blue) and negative (red). (B) Similarly, potentials are shown for apolar sites paired with four site types: apolar (black), polar (cyan), positive (blue) and negative (red). (C) Potentials are shown for polar sites paired with site types: polar (cyan), positive (blue) and negative (red). (D) Lastly, potentials are shown for three site pairings: positive-positive (blue), positive-negative (red) and negative-negative (pink). All 15 potentials have softer core repulsions than an LJ potential. Model parameters are available upon request. (0.48 MB TIF) [file pcbi.1000827.s003.tif]

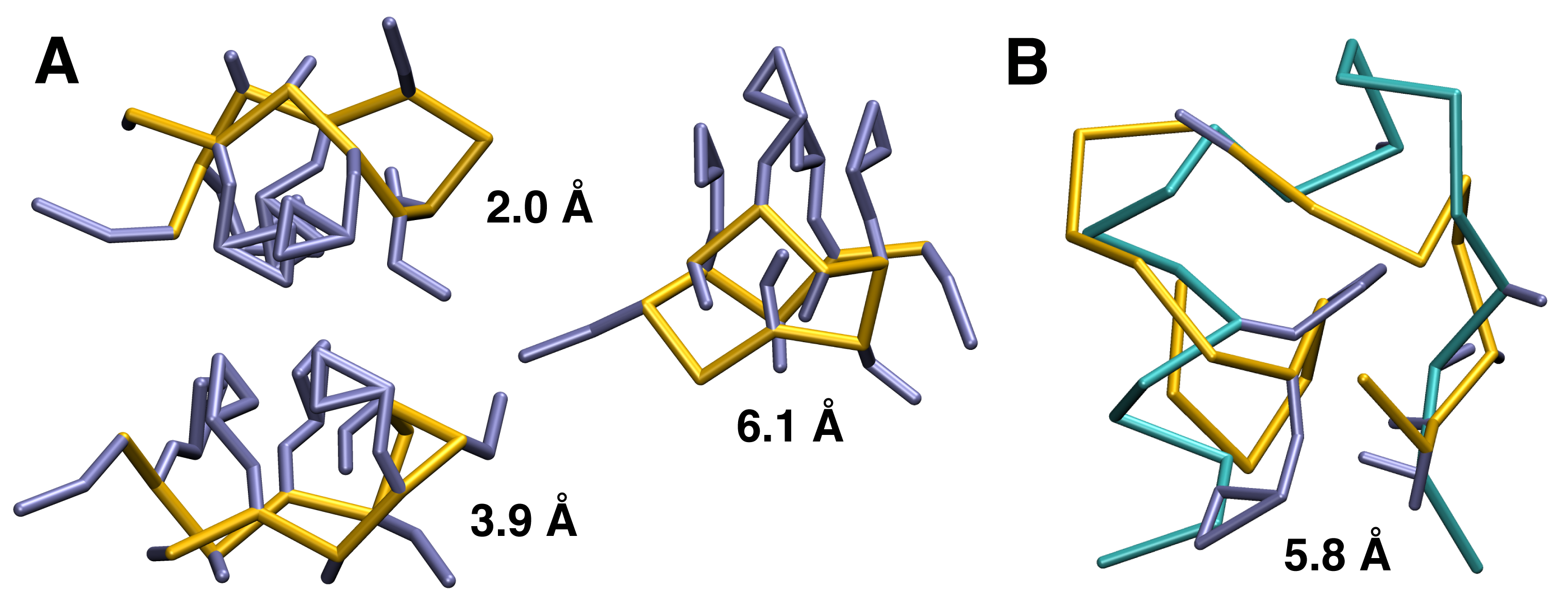

Supplement: Figure S4 — Energy minima in CG-REMD folding simulations. Representative snapshots are shown corresponding to the three energy minima of Trpzip (A) and the global minimum of Trp-cage (B), along with their Cα RMSD from the native structure. Backbone Cα-traces are shown in yellow along with Trp, Pro and other sidechains in blue. The Trp-cage native structure is shown in cyan (backbone). Snapshots were obtained from CG-REMD folding simulations collected at 0.6 (see Figure 5). (1.99 MB TIF) [file pcbi.1000827.s004.tif]

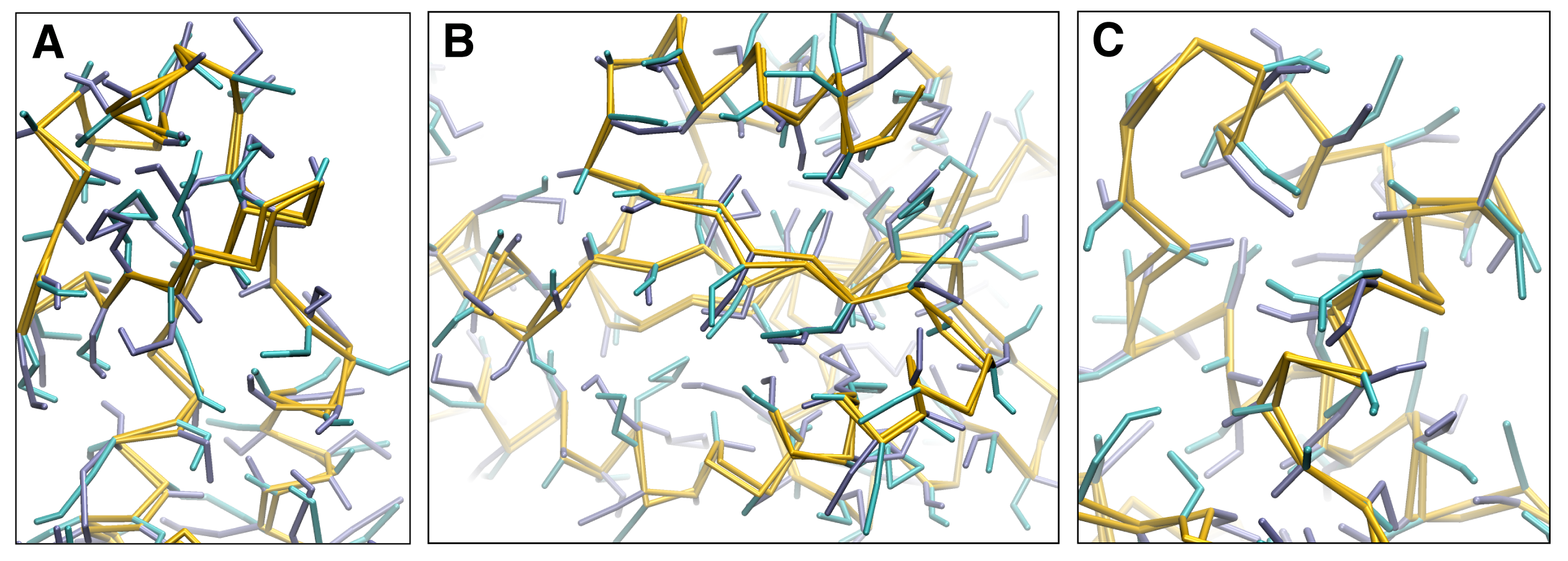

Supplement: Figure S5 — Sidechain prediction with CG-REMD. The final configuration is shown after 80 ns of CG-REMD at 0.6 with the backbone (yellow) fixed in open AdK. Sidechain sites (blue) exhibited final RMSDs of 2.9 Å, 3.1 Å and 2.9 Å from their starting native positions (cyan) for the LID (A), CORE (B) and NMP (C) domains, respectively. (2.35 MB TIF) [file pcbi.1000827.s005.tif]
